# A novel approach for solving travelling thief problem using enhanced simulated annealing

**DOI:** 10.7717/peerj-cs.377

**Published:** 2021-03-16

**Authors:** Hamid Ali, Muhammad Zaid Rafique, Muhammad Shahzad Sarfraz, Muhammad Sheraz Arshad Malik, Mohammed A. Alqahtani, Jehad Saad Alqurni

**Affiliations:** 1 Department of Computer Science, National Textile University, Faisalabad, Pakistan; 2 Department of Computer Science, National University of Computer and Emerging Sciences, Islamabad, Pakistan; 3 Department of Information Technology, Government College University, Faisalabad, Faisalabad, Pakistan; 4 Department of Computer Information Systems, College of Computer Science and Information Technology Imam Abdulrahman Bin Faisal University, Dammam, Saudi Arabia; 5 Department of Education Technologies, College of Education, Imam Abdulrahman Bin Faisal University, Dammam, Saudi Arabia

**Keywords:** Travelling thief problem, Optimization, Heuristics, Evolutionary algorithm, Enhanced simulated annealing, Traveling salesman problem, Knapsack problem

## Abstract

Real-world optimization problems are getting more and more complex due to the involvement of inter dependencies. These complex problems need more advanced optimizing techniques. The Traveling Thief Problem (TTP) is an optimization problem that combines two well-known NP-Hard problems including the 0/1 knapsack problem and traveling salesman problem. TTP contains a person known as a thief who plans a tour to collect multiple items to fill his knapsack to gain maximum profit while incurring minimum cost in a standard time interval of 600 s. This paper proposed an efficient technique to solve the TTP problem by rearranging the steps of the knapsack. Initially, the picking strategy starts randomly and then a traversal plan is generated through the Lin-Kernighan heuristic. This traversal is then improved by eliminating the insignificant cities which contribute towards profit adversely by applying the modified simulated annealing technique. The proposed technique on different instances shows promising results as compared to other state-of-the-art algorithms. This technique has outperformed on a small and medium-size instance and competitive results have been obtained in the context of relatively larger instances.

## Introduction

Optimization problems are handling more efficiently on a daily basis, and this aspect considered that these problems are becoming more complex. These several optimization real-world problems (Laporte, 1992; Kellerer, Pferschy & Pisinger, 2004) are interacting with each other. Moreover, problems are complex to solve, especially those which are not purely independent. Real-life optimization problems usually contain several problems that interrelate with each other. In order to resolve these problems, it is important to realize and deal with these interactions. So far, the research literature is efficient in systematic approaches for dealing with such problems that are interdependent. The Travelling Thief Problem (TTP) is one of the problems which interacts with two well-known NP-hard sub problems as the Travelling Salesman Problem (TSP) (Laporte, 1992) and the 0/1 Knapsack Problem (KP) (Kellerer, Pferschy & Pisinger, 2004). The Travelling Thief Problem was introduced in 2013 by Bonyadi, Michalewicz & Barone (2013). It is a relatively new optimization problem as it is the combination of two different optimization problems. This problem is introduced by researchers when they are trying to compare different metaheuristics that take place in the perspective of NP-hard optimization problems. During this research, they found a gap between these benchmarks and real-world problems.

In the TTP, a person (thief) travel from a city and make a round trip trough the available cities. There are multiple items are present in each city, which have some weight and value, The thief has a knapsack with a limited capacity and wants to collect the items available in different cities, which maximizes his profit without exceeding the knapsack capacity (Wachter, Thesis & Der Arbeit, 2015). The problem is that as the thief travels and collects the items, his speed decreases because of the increase of weight of the knapsack. This problem leads to longer travel time, which ultimately increases the rent of knapsack and ride. This problem limits the overall profit of the thief.

In this research, we rearrange the steps of TTP. Initially, picking strategy starts randomly and then a traversal plan is generated through the Lin-Kernighan heuristic (Applegate, Cook & Rohe, 2003). Each city has different items that contain a value and a weight, but keep in mind that items can be placed in more than one city. These items can be placed in a knapsack during the traversal plan but it must be noted that knapsack capacity should be limited and cannot be exceeded by given capacity. The goal of this optimization problem is to achieve maximum profit in a limited time as 600 s, which is the standard time of TTP, and without exceeding the knapsack capacity.

By collecting the items, a thief tries to pick those items which are more valuable and less in weight in order to achieve maximum gain. However, note that by collecting the items, the velocity of a thief will decrease as the empty knapsack capacity increases by collecting the items, which affect the total time of the tour. Moreover, the thief has to pay rent for his knapsack which is directly proportional to the tour time. Two sub problems are independent of each other because when more items are picked, it affects the thief by slowing down velocity, and the knapsack is rented so it will affect the important travel time (Yafrani & Ahiod, 2018).

In this research, we have to solve the traveling thief problem more efficiently by planning a tour and a picking plan. This problem has been solved by different techniques, but they need to improve more because it is a hybrid problem (Wu et al., 2017; Moeini, Schermer & Wendt, 2017). To solve this problem, researchers firstly make a tour plan using all cities and secondly decide the picking plan, but in this paper we have proposed a different approach in which we first make a picking plan and then arrange a tour in which the overall cost of the problem is minimized. According to our picking plan, we dismissed the cities from which we have not pick any item and considered only those cities from which we have to pick items. So, this planned tour gave the results much profitable.

The TTP is a benchmark problem intended to address concerns presented in that there is a gap between theory and practice in the field of meta heuristics for combinatorial optimization problems. It is claimed that the definition of complexity is the main difference between the benchmark problems used in theoretical work, and the real-world-problems to which the results are intended to be applied in practice. For the benchmark problems, it is claimed, there is a tendency to equate complexity with size for example, a number of cities for the TSP while real-world problems usually include additional sources of complexity, such as the interdependence of components problems, as intentionally featured by the TTP (Mei, Li & Yao, 2016).

So, for the TTP, there exist different kinds of heuristic solvers that feature different levels of communication, and there appears to be an expectation that such communication is necessary (Mei et al., 2015). As new algorithms are developed, it would be interesting to see how well this expectation is met. This research performs a novel technique to solve the optimization problem in which real-world problems are modeled by interconnecting with a different problem. To solve such problems, the main challenging task is the complexity which we should reduce to solve problems, as one problem influencing the other problem and vice versa (Bonyadi et al., 2019). Subsequently, the computed results of instances include the large range with the different feature which start from a limited or few numbers of cities with limited items and knapsack to a large set of cities with a large number of items and include the large knapsack capacity (Polyakovskiy et al., 2014). While the small set of instances and medium-size instances are solved soon and give an optimal solution but large instances remain unsolved for a number of years. These instances are basically introduced in the TSP problem and further mold it with knapsack parameters. TSPLIB library is used from which these instances are taken to solve the problem (Reinelt, 1991).

The rest of the article is organized as follows: In “Background”, we briefly discuss the basic concepts of traveling salesman problem, knapsack problem, and traveling thief problem. “Methodology” describes the methodology of the proposed approach. The experiments and results are presented in “Results and Discussions”. “Conclusion” concludes the article.

## Background

### Travelling salesman problem

Travelling salesman problem is considered as the well-known NP-Hard problem which contains *N* cities and a traveling salesman make a tour where all cities have to be visited exactly once (Nallaperuma et al., 2013). The salesman person starts a tour from the starting point and also ended at the same starting point after visiting all the cities. The goal of this problem is to minimize travel time considered as a traveling cost. Moreover, there is no time limit and no velocity mentioned in this problem. There are different parameters of traveling salesman problem with a single objective to minimize the cost. Moreover, all the detailed parameter are discussed and explain more clearly for the understand-ability of TSP: There are *N* number of cities, where city set is, *X* = {1, 2,…, *n* }, *D*_*i*,__*j*_ is a distance matrix which finds the distance from one city to all other cities, there are many items in each city, velocity is denoted by *V* and it remains constant during the tour, Tour *X*_*i*_ where city *i* contains *n* number of items containing all cities in the order in which they can be visited, the travel time *t* between city *X*_*i*_ and *X*_*i*+1_, ∀*i* = 1,2,…,*n* is calculated through Objective function *f*(*X*) is considered as:

(1) }{}{\rm Min}\;f(X) = \sum\limits_{i = 1}^n ({t_{{x_i},{x_{i + 1}}\quad {\rm mod}\; n}})

### Knapsack problem

The knapsack problem is an NP-hard problem that we can solve by different optimization techniques. The knapsack has a limited capacity which is mentioned with symbol C. Moreover, this problem contains the items which have different weight and values. Weight and value are denoted by *w*_*i*_ and *p*_*i*_ respectively and items are denoted by *i*. Items are packed into the knapsack according to the capacity by focusing on the value and weight of each item. Here, this problem has to gain maximum profit by selecting the optimal combination of items (Stolk et al., 2013).

Number of Items can be considered as ( *i* = 1 to *m* ), *P*_*i*_*εR* value of an item, weight *w*_*i*_*εR* weight of items, the limited capacity of knapsack is *C* , Itemset \overrightarrow Z which contains or packed the items in the knapsack. \overrightarrow Z = \{ {z_1},{z_2}, \ldots ,{z_m}\}; *z*_*i*_*ε*{0;1}; that is, *z*_*i*_ = 1 item *i* is packed and *z*_*i*_ = 0 item *i* is not packed, Objective function of the KP is to maximize the total value of items selected without exceeding the limit of the knapsack capacity which are formulated below.

(2) \text{Weight\;Constraint} = \sum\limits_{i = 1}^m ({w_i}{z_i} \le C)

(3) }{}{\rm Maximize}\;g(\overrightarrow Z ) = \sum\limits_{i = 1}^m {p_i}{z_i},\;{\overline {\rm z} _i} = \{ {z_1},{z_2}, \ldots ,{z_m}\}

### Travelling thief problem

The benchmark solution set is considered to solve the TTP, as the authors of the TTP benchmark solution set also presented many approaches with different variants to solve this optimization problem (Polyakovskiy et al., 2014). At first, this problem was drive by Bonyadi et al. (2014) combining two well-known NP-hard problems and proposed two models to solve TTP. These models are proposed but most of the researchers focus on the first model.

Different researchers use different approaches to solve this problem that is, a random local search (Deb & Sinha, 2010) is applied in early and evolutionary algorithms with a simple (1+1) approach (Yafrani & Ahiod, 2017). Some optimization algorithms like ACO and Genetic algorithms (NSGA) (Laszczyk & Myszkowski, 2019) are also applied but the efficiency of approaches is limited to specific scenarios (Wagner, 2016). There is only one objective to maximize the profit in the first model and two new parameters are also introduced to make these sub-problems interdependent (Yafrani et al., 2018). Therefore Faulkner only computes the fitness after multiple items are added and backtrack if the score became worse (Faulkner et al., 2015; Bonyadi et al., 2019). The traveling speed is related to the knapsack weight and knapsack rent which is paid and increased R per unit time. Two objectives are considered in the second model as maximizing the total profit and minimizes the time and cost by adding the three parameters (Deb & Sinha, 2009). Moreover, many other variants are added in traveling thief problem to solve this by molded the problem in other dimensions that is, Multiple Knapsack Problem (Lalami et al., 2012), multi-objective knapsack (Bazgan, Hugot & Vanderpooten, 2009), fractional knapsack (Ishii, Ibaraki & Mine, 1977), bi-level knapsack (Chen & Zhang, 2013), etc. After this, the benchmark is designed with different algorithms to solve TTP but these are the simple techniques to verify this problem (Polyakovskiy et al., 2014).

Now we formulate the TTP which is the combination of two well-known bench mark problems: Knapsack problem and Travelling salesman problem. We consider as,

- *N* (cities),
- *m* (items) scattered in the *N* cities,
- Each item *i* having a weight *w*_*i*_ and a value *p*_*i*_
- *W* (knapsack capacity) is the maximum capacity of the knapsack,
- *V*_max_ = 1 is the maximum velocity,
- *V*_min_ = 0.1 is the minimum velocity and
- *D* = distance matrix.

The distance matrix *D* is calculated to find the cost from each city to another city where the star symbol represents that no path is found.

}{}D = \left( {\matrix{ -  5  *  *  4 \cr 5  -  6  3  5 \cr *  6  -  8  6 \cr *  3  8  -  7 \cr 4  5  6  7  -  } } \right)

- The (*p*, *w*) for available items (*I_i_*) are: *I*_1_ = (20,1), *I*_2_ = (25,2), *I*_3_ = (60,2), *I*_4_ = (30,3), *I*_5_ = (50,2), *I*_6_ = (40,3),
- Availability (*A_i_*) of each item in cities is: *A*_1_ = 2,5, *A*_2_ = 4, *A*_3_ = 3,4, *A*_4_ = 4, *A*_5_ = 2, *A*_6_ = 5,
- *R* = rent per unit travel time.

**Figure 1:**
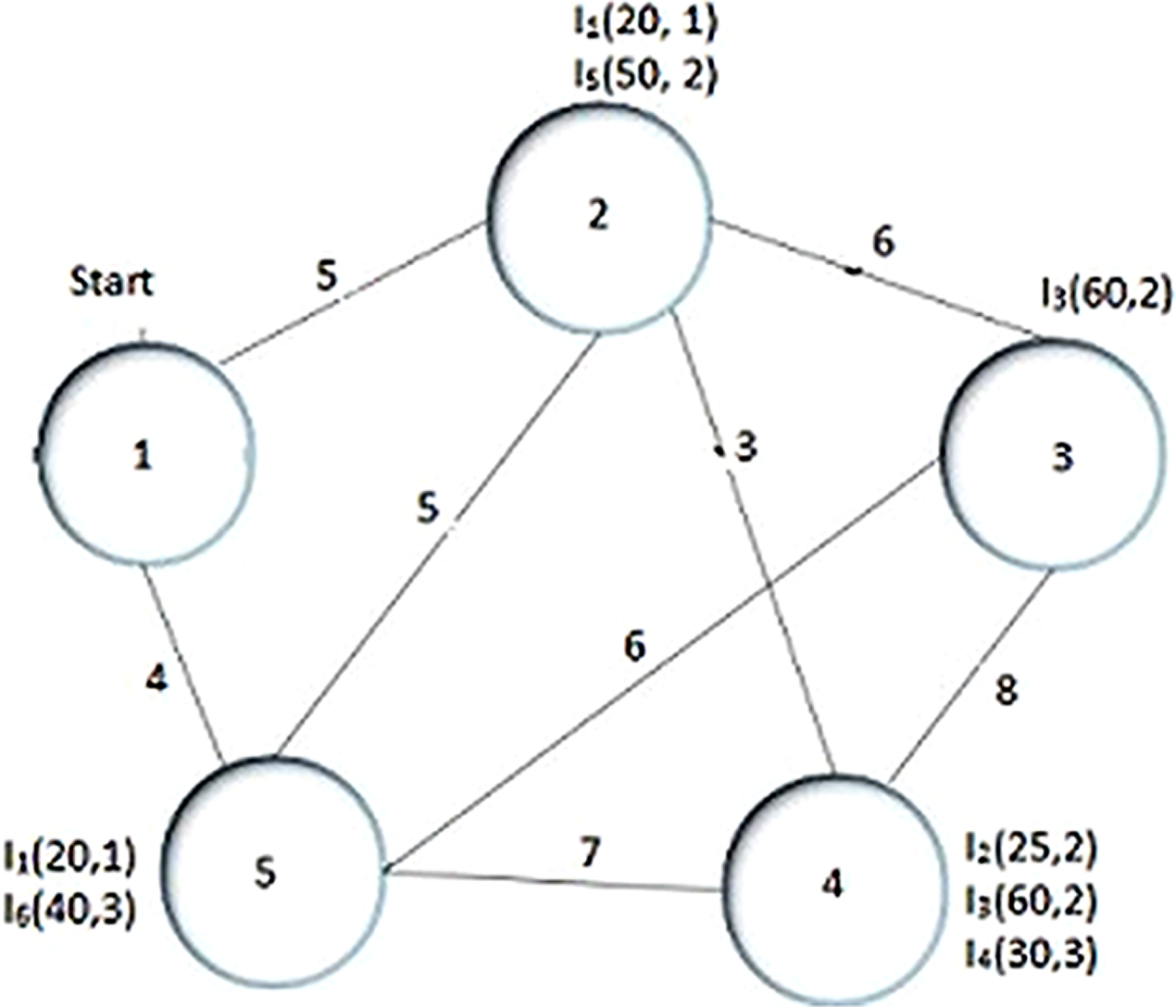
Example of TTP.

In the given example as shown in Fig. 1 the thief starts tour from node 1 moving to node 2 or 5, where the distance between these nodes are 5 and 4 respectively. The current weight of the knapsack (*W*_*c*_) is 0, thus, *V*_*c*_ = *V*_max_ = 1, which results in *t*_1,2_ (cost/time taken from city 1 to city 2) is 5. Equation (4) is used to calculate the current speed of thief as

(4) }{}{V_c} = {V_{\max}} - \left({W_c}\displaystyle{{{V_{\max}} - {V_{\min}}} \over W}\right)

By this equation, we find the current speed of the thief. The speed of the thief fluctuates according to the weight of knapsack. So, the speed of the thief and knapsack capacity are inversely proportional to each other as the knapsack is filled, the thief becomes slow. After the compilation of all process, the solution of proposed example is represented as *x* = 1, 3, 2, 4, and 5 which shows the cities for travel in a proper order and *z* = 0, 5, 0, 3, and 1 that indicates the items which are picked from city according to the index, for example, item *I*_5_ is packed from city 2, *I*_3_ is packed from city 4 and *I*_1_ is packed from city 5 respectively. The goal of this problem is to find the maximum profit *G*(*x*, *z*) by using the value }{}g(\overrightarrow Z ) produced by picking plan using Eq. (3) and the rent *R* × *f*(*X*) produced using Eq. (1) as

(5) }{}G(x,z) = g(\overrightarrow Z ) - R \times f(X)

## Methodology

**Figure 2:**
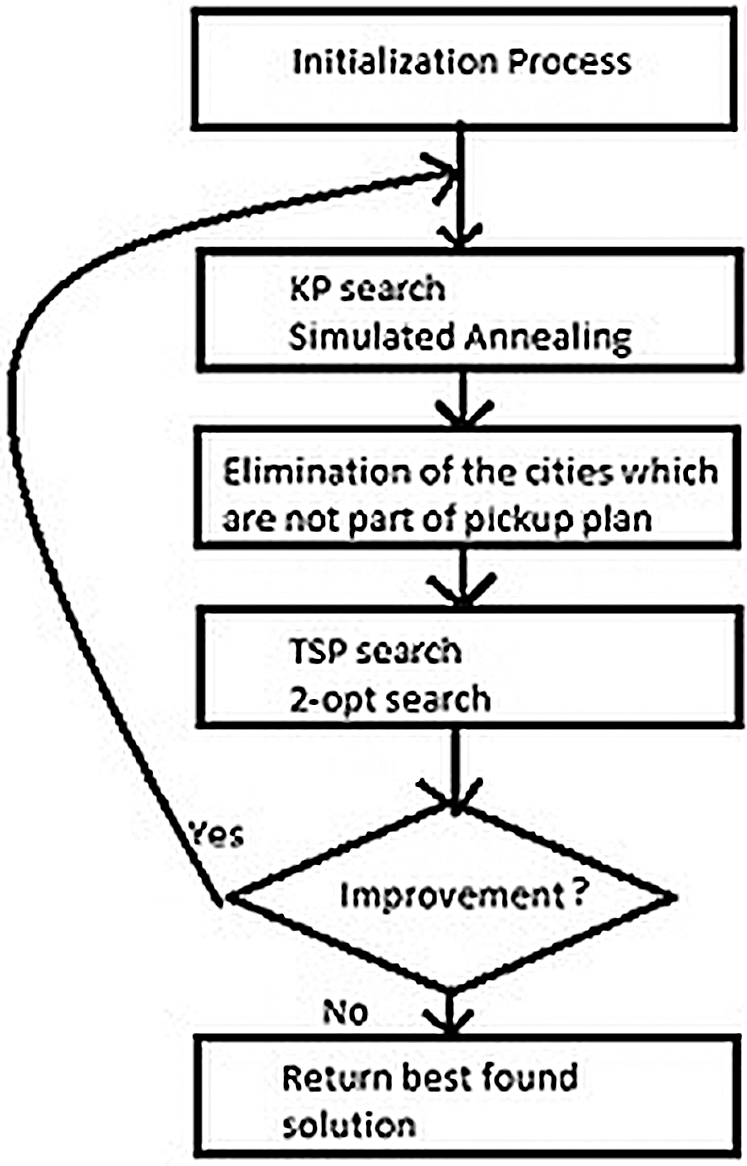
ESA simplified flowchart.

This section includes the methodology of TTP in which we initialize the solution strategy to tackle the traveling thief problem. We proposed an efficient technique to solve the TTP problem by rearranging the steps used in this problem. Initially, picking strategy starts randomly and then a traversal plan is generated through Lin-Kernighan heuristic. This traversal is then improved by eliminating the insignificant cities by applying the modified simulated annealing technique. The knapsack is filled by picking the most profitable items and used three fitness functions to maximize the profit (Martins et al., 2017). The Extended Simulated Annealing (ESA) is summarized in the flow chart in Fig. 2.

### Initialization

In this stage we initialize the basic parameters and randomly produces the solution by picking the items from the different cities and generate the tour.

#### KP search

After the initialization process, we select the items with the help of objective function and then eliminate the cities from which we do not pick any item. This process saves our travel time and cost through which our profit will increase at a maximum level. The limitation of this technique is highlighted when one city *X*_*i*_ is only connected to another city *X*_*i*+1_, but we have deleted the city *X*_*i*_ because we have no interest in the items of this city, but their corresponding city *X*_*i*+1_ is the only city from which we have to pick an item but we have deleted the city *X*_*i*_, so after this we cannot reach at *X*_*i*+1_ in this situation. So, at that stage, we may get the infinity value as a profit.

The simulated Annealing approach is used for this problem. The following parameters of SA are used:

- The absolute temperature *T*_abs_, set to 1.
- The initial temperature *T*_0_, set to 100.
- temperature cooling parameter α, set to 0.95.
- The number of iterations depends upon the size of the instances.
- *m* (items) scattered in the *N* cities,

#### TSP search

In TSP search, we finds the best tour *x* by using the previous picking plan *z* to get profit. Further, we also focused on the travel cost instead of profit, because the profit also depends on the travel cost by eliminating it from gain profit. So, we also try to minimize the total traveling cost. Similarly, the picking plan is also improved by comparing the current picking with the previous plan and considered the best plan for further implementation.

## Results and discussions

The results are computed on different instances, as discussed in Table 1. Moreover, this section graphically represent the results produced by all the algorithms using instances individually and provide a comparison in Table 2 as profit and time of each instance.

**Table 1:** Parameters of instances.

Instances	Cities	Items	Item Factor	Knapsack capacity	Renting ratio
*eil*51_50_*bsc*	51	50	1	4,029	4.44
*eil*51_50_*un*	51	50	1	6,679	12.95
*eil*51_150_*usw*	51	150	3	82,197	57.66
*eil*76_225_*bsc*	76	225	3	19,571	27.52
*eil*76_750_*un*	76	750	10	336,137	279.05
*eil*76_375_*usw*	76	375	5	34,244	23.10
*kro*100_99_*bsc*	100	99	1	8,332	0.30
*kro*100_990_*un*	100	990	10	450,400	8.36
*kro*100_990_*usw*	100	990	10	632,868	7.67
*ch*130_129_*bsc*	130	129	1	10,329	0.94
*ch*13_129_*un*	130	129	1	5,937	1.52
*ch*130_1290_*usw*	130	1,290	10	824,671	35.49
*u*159_158_*bsc*	159	158	1	13,006	0.20
*u*159_1580_*un*	159	1,580	10	717,438	7.15
*u*159_790_*usw*	159	790	5	721,446	3.57
*a*280_279_*bsc*	280	279	1	25,936	5.61
*a*280_279_*un*	280	279	1	12,718	5.93
*a*280_279_*usw*	280	279	1	127,391	14.77
*u*574_*n*573_*bsc*	574	573	1	51,126	1.05
*u*574_*n*573_*un*	574	573	1	25,737	1.01
*u*574_*n*573_*usw*	574	573	1	52,326	0.48
*u*724_723_*bsc*	724	723	1	64,888	0.87
*u*724_2169_*un*	724	2,169	3	196,778	4.54
*u*724_3615_*usw*	724	3,615	5	1,650,549	11.53
*dsj*1000_*n*2997_*bsc*	1,000	2,997	3	260,213	0.01
*dsj*1000_*n*999_*un*	1,000	999	1	45,302	0.00
*dsj*1000_*n*999_*usw*	1,000	999	1	91,232	0.00
*rl*1304_3909_*bsc*	1,304	3,909	3	338,470	0.86
*rl*1304_6515_*un*	1,304	6,515	5	297,425	1.70
*rl*1304_13030_*usw*	1,304	6,515	5	4,759,562	5.78
*fl*1577_*n*1576_*bsc*	1,577	4,728	3	137,031	4.04
*fl*1577_*n*4728_*un*	1,577	4,728	3	214,859	13.90
*fl*1577_*n*1576_*bsc*	1,577	1,576	2	431,777	6.65
*d*2103_*n*2102_*bsc*	2,103	2,102	1	361,309	2.48
*d*2103_*n*6303_*un*	2,103	6,303	3	862,922	8.74
*d*2103_*n*2102_*usw*	2,103	2,102	1	191,954	0.92
*pcb*3038_*n*3037_*bcs*	3,038	3,037	1	1,053,070	4.00
*pcb*3038_*n*3037_*un*	3,038	3,037	1	137,833	1.43
*pcb*3038_*n*3037_*usw*	3,038	3,037	1	277,324	0.71
*fnl*4461_*n*4460_*bcs*	4,461	4,460	1	774,300	2.48
*fnl*4461_*n*22300_*un*	4,461	22,300	5	1,012,959	8.59
*fnl*4461_*n*4460_*usw*	4,461	4,460	1	407,280	0.90
*pla*7397_*n*7396_*bsc*	7,397	7,396	1	1,926,164	0.04
*pla*7397_*n*7396_*un*	7,397	7,396	1	338,070	0.02
*pla*7397_*n*7396_*usw*	7,397	7,396	1	675,372	0.01
*rl*11849_*n*59240_*bsc*	11,849	59,240	5	1,020,234	0.72
*rl*11849_*n*11848_*un*	11,849	11,848	1	537,079	0.87
*rl*11849_*n*35544_*usw*	11,849	35,544	3	12,983,176	4.52
*usa*13509_40524_*bsc*	13,509	1,576	3	3,496,473	0.10
*usa*13509_13508_*un*	13,509	13,508	1	613,099	0.04
*usa*13509_40524_*usw*	13,509	40,524	3	18,502,846	0.26
*brd*14051_42150_*bsc*	14,051	42,150	3	3,642,020	4.93
*brd*14051_140500_*un*	14,051	140,500	10	64,046,502	56.24
*brd*14051_70250_*usw*	14,051	70,250	5	25,660,440	16.87
*d*15112_15111_*bsc*	15,112	15,111	1	1,303,355	0.51
*d*15112_151110_*uc*	15,112	151,110	10	68,850,446	17.61
*d*15112_15111_*usw*	15,112	15,111	1	6,899,608	1.24
*d*18512_*n*55533_*bsc*	18,512	55,533	3	23,888,995	19.59
*d*18512_*n*55533_*un*	18,512	55,533	3	2,523,961	6.15
*d*18512_*n*185110_*usw*	18,512	185,110	10	169,039,320	58.15
*pla*33810_101427_*bsc*	33,810	101,427	3	8,731,411	0.08
*pla*33810_338090_*uc*	33,810	338,090	10	153,960,049	0.94
*pla*33810_169045_*usw*	33,810	169,045	5	154,369,589	0.47

**Table 2:** Comparison with existing techniques.

Instances	S5			CS2A/CS2SA R			ESA		
	Mean	std	T	Mean	std	T	Mean	std	T
*eil*76_255_*bsc*	3,742	0	600	3,842	0.25	600	18,835	0	108
*eil*76_750_*un*	88,136	0	600	88,099	1.2	600	155,765	0	159
*eil*76_375_*usw*	22,188	0	600	22,032	0.36	600	59,072	0.54	345
*kro*100_99_*bsc*	4,659	0	600	155,878	1.07	600	10,502	0	63
*kro*100_990_*un*	155,540	0	600	155,878	2.42	600	166,617	0	99
*kro*100_990_*usw*	42,595	0	600	43,712	0.53	600	41,411	0	25
*ch*130_129_*bsc*	9,250	0	600	9,239	0.42	600	8,3197	0.73	45
*ch*130_129_*un*	201,174	1.27	600	20,6461	0.72	600	51,936	0	202
*ch*130_1290_*usw*	61,061	0.85	600	60,864	0	600	62,639	0	84
*u*159_158_*bsc*	8634	0	600	8,560	0	600	16,385	0.11	210
*u*159_1580_*un*	242,495	0	600	249,911	1.03	600	159,542	0.42	301
*u*159_790_*usw*	57,618	0.31	600	60,377	0	600	229,648	0	253
*a*280_279_*bsc*	18,406	0.02	600	1,804	0.13	600	22,787	0.1	600
*a*280_279_*un*	429,000	0.01	600	421,713	0.84	600	7,8357	0.21	171
*a*280_279_*usw*	109,921	0	600	114,087	0	600	126,725	0	593
*u*574_*n*573_*bsc*	26,933	0.14	600	26,173	1.06	600	515,842	0.1	88
*u*574_*n*573_*un*	966,344	0.05	600	953,997	1.05	600	25,670	0.41	44
*u*574_*n*573_*usw*	254,770	0.05	600	248,584	0.4	600	162,443	0.12	180
*u*724_723_*bsc*	50,316	0.12	600	49,713	0.56	600	664,583	0	115
*u*724_2169_*un*	1,188,364	0.35	600	1,197,819	1.11	600	20,284	0.12	150
*u*724_3615_*usw*	305,977	0.14	600	809,636	0.36	600	1,331,568	0.46	260
*dsj*1000_*n*2997_*bsc*	137,631	0.12	598	144,219	0	600	1,195,955	0	600
*dsj*1000_*n*999_*un*	1,479,618	0.59	600	1,468,858	2.21	600	456,720	0	185
*dsj*1000_*n*999_*usw*	342,189	0.1	599	339,136	0.74	600	500,563	0.5	336
*rl*1304_3909_*bsc*	80,066	0.94	598	75,699	1.38	600	1,705,291	0	300
*rl*1304_6515_*un*	2,184,853	0.86	600	2,198,643	1.41	600	1,247,295	0.4	310
*rl*1304_13030_*usw*	575,102	0.21	599	585,600	0	600	1,706,246	0.58	280
*fl*1577_*n*1576_*bsc*	92,343	1.25	597	84,590	1.64	600	1,808,925	0	236
*fl*1577_*n*4728_*un*	2,470,917	1.62	598	250,5291	0	600	2,481,549	0.63	405
*fl*1577_*n*1576_*usw*	607,247	.36	599	636,425	0	281	765,587	0.35	600
*d*2103_*n*2102_*bsc*	120,642	0.2	597	118,845	1.4	305	1,012,468	1.5	260
*d*2103_*n*6303_*un*	3,392,172	1.15	599	337,3781	0	75	2,256,660	1.24	71
*d*2103_*n*2102_*usw*_*z*	853,587	0.15	600	842,520	0	301	1,617,039	0	600
*pcb*3038_*n*3037_*bcs*	160,006	0.15	598	145,338	1.65	600	355,982	0	600
*pcb*3038_*n*3037_*un*	457,374	0.2	596	461,295	0	148	516,919	0	164
*pcb*3038_*n*3037_*usw*	1,179,510	0.09	597	1,193,738	0	395	137,397	0	49
*fnl*4461_*n*22300_*un*	6,554,497	0.11	592	6,545,335	0.67	242	5,290,096	1.54	201
*fnl*4461_*n*4460_*usw*	1,625,856	0.18	596	162,8414	0	157	6,263,961	1.51	49,881
*pla*7397_*n*7396_*bsc*	395,156	0.01	591	315,154	9.95	600	4,230,637	2.04	600
*pla*7397_*n*7396_*un*	14,239,601	0.79	569	13,197,756	9.37	242	15,350,548	1.7	521
*pla*7397_*n*7396_*usw*	4,371,424	3.87	591	3,713,314	0	383	1,571,873	0.98	498
*rl*11849_*n*59240_*bsc*	707,190	0.29	591	657,842	0.46	600	1,343,435	0	502
*rl*11849_*n*11848_*un*	18,314,650	0.29	582	18,504,773	1.22	600	11,885,976	0	459
*rl*11849_*n*35544_*usw*	4,371,424	0.16	587	3,713,314	0	600	5,949,523	0	217
*usa*13509_1576_*bsc*	809,607	0.24	582	682,268	3.7	600	3,097,233	0	288
*usa*13509_13508_*un*	25,918,971	0.72	568	26,436,928	2.54	595	21,288,809	1.15	350
*usa*13509_40524_*usw*	7,818,124	0.4	579	8,115,168	0	600	15,230,335	2.21	249
*brd*14051_42150_*bsc*	875,018	029	586	802,424	0.53	600	1,242,896	0	174
*brd*14051_140500_*un*	23,826,394	0.61	577	23,908,555	1.09	600	19,023,469	0.58	600
*brd*14051_70250_*usw*	6,552,858	0.5	587	6,654,162	0.95	600	12,764,392	1.41	302
*d*15112_15111_*bsc*	939,790	0.47	585	871,284	0.67	600	7,330,511	2.08	507
*d*15112_151110_*un*	26,211,266	1.31	577	27,183,354	0	600	28,822,404	0	600
*d*15112_15111_*usw*	6,991,440	0.87	578	7,606,856	1.32	600	12,764,392	0	600
*d*18512_*n*55533_*bsc*	1,072,308	0.21	582	964,757	0.62	554	3,899,170	0	555
*d*18512_*n*55533_*un*	27,427,135	0.38	569	27,823,470	0.01	600	15,734,969	0	234
*d*18512_*n*185110_*usw*	7,257,709	0.35	600	7,579,996	1.06	600	2,967,318	1.54	600
*pla*33810_101427_*bsc*	1,870,330	0.78	572	1,778,827	1.83	600	9,480,228	1.55	600
*pla*33810_338090_*un*	57,967,446	0.73	541	58,106,820	0.5	600	29,618,079	3.21	600
*pla*33810_169045_*usw*	15,574,552	0.39	566	15,704,051	0.01	600	16,970,318	3.60	600

The Table 1 demonstrates all parameters of each instance in which a number of cities and the number of items is included. Factor items are the number of items per city and note that each item has its own profit and weight. Further, the first node which is the starting node has no item in all instances. The knapsack capacity is also mentioned in the Table 1, which is the limited capacity of the knapsack and different for each instance.

In this research, the benchmark dataset included thousands of files. These files are further divided into three categories based on their sizes as small, medium and large instances. These sizes are described in different ranges which are based on the number of cities and the number of items per city. The small size included the range of files up to 1,000 cities and included all types of files that is, bounded correlated strongly (bsc), uncorrelated (un) and uncorrelated with similar weights (usw).

Moreover, Table 2 represents the overall result of the instances by comparing the ESA with two existing algorithms. It includes the mean value which is the profit of the thief and the execution time of each instance in seconds as 600 is the maximum time to execute the instance file.

In Table 2, the proposed technique is compared with two existing techniques the memetic algorithm, hill climbing, and simulated annealing technique as a hybrid (Yafrani & Ahiod, 2016; Lourenço, Pereira & Costa, 2016). The first column of the table represents the instances file individually and then each column mentioned the mean value or the objective value of instances, standard deviation (std), as well as the time (T) in seconds. Average result is presented in Table 2 after executing 10 times per input file.

Here, clearly shown that the proposed techniques perform very well with respect to mean value and the time (Wagner et al., 2018). Further, by comparing these techniques the small instances show good results as they are shown in the first twenty one rows. The proposed technique is very efficient as in these small instances the minimum time period is calculated 25 s which are very less. This is happened just because of eliminating the cities, the time travel is reduced to a minimum and generate a tour. Sometimes, it may increase to the maximum time limit as shown in a 280 bounded strongly correlated instances but this time reached in only a few instances.

Moreover, the medium-size instances also perform better according to their range, it includes the instances from rl1304 to brd14051. As shown clearly in Table 2 that the last file of bounded strongly correlated perform their computation in 174 s and the existing techniques compute the results in 586 and 600 s respectively. Subsequently, in all medium-size instances, the performance is better with respect to both time and objective value.

The results of large instances also perform better and it includes the only nine instance files. In these instances the existing techniques perform better in only three instances and on the other six instances, the proposed algorithm is very effective. For the time comparison of large instances, the existing techniques are better to compute the file but the difference is minor. So, this approach concluded that it performs significantly well for small and medium sizes and compatible with the large size instance. By eliminating the non-benefit cities from the tour this technique differs on the large size and gives better results. There are some graphical representation of the results of all the categories is given below.

**Figure 3:**
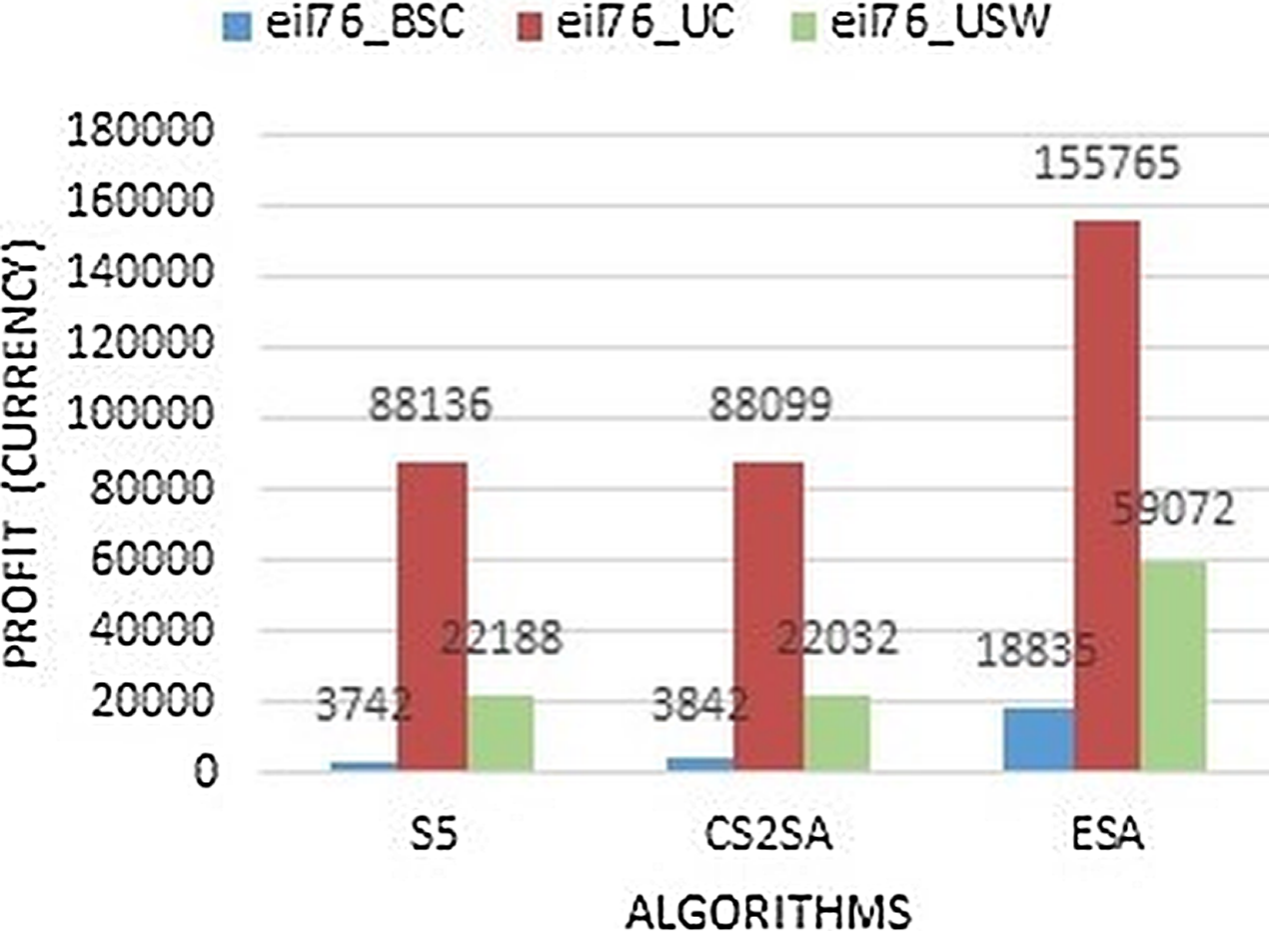
Result comparison of instance eil76.

The Fig. 3 shows that our technique performs better in all types of instances as it gains 18,835, 155,765 and 59,072 of BSC, UN, and USW respectively. So the performance of ESA is much better in eil76 instance.

**Figure 4:**
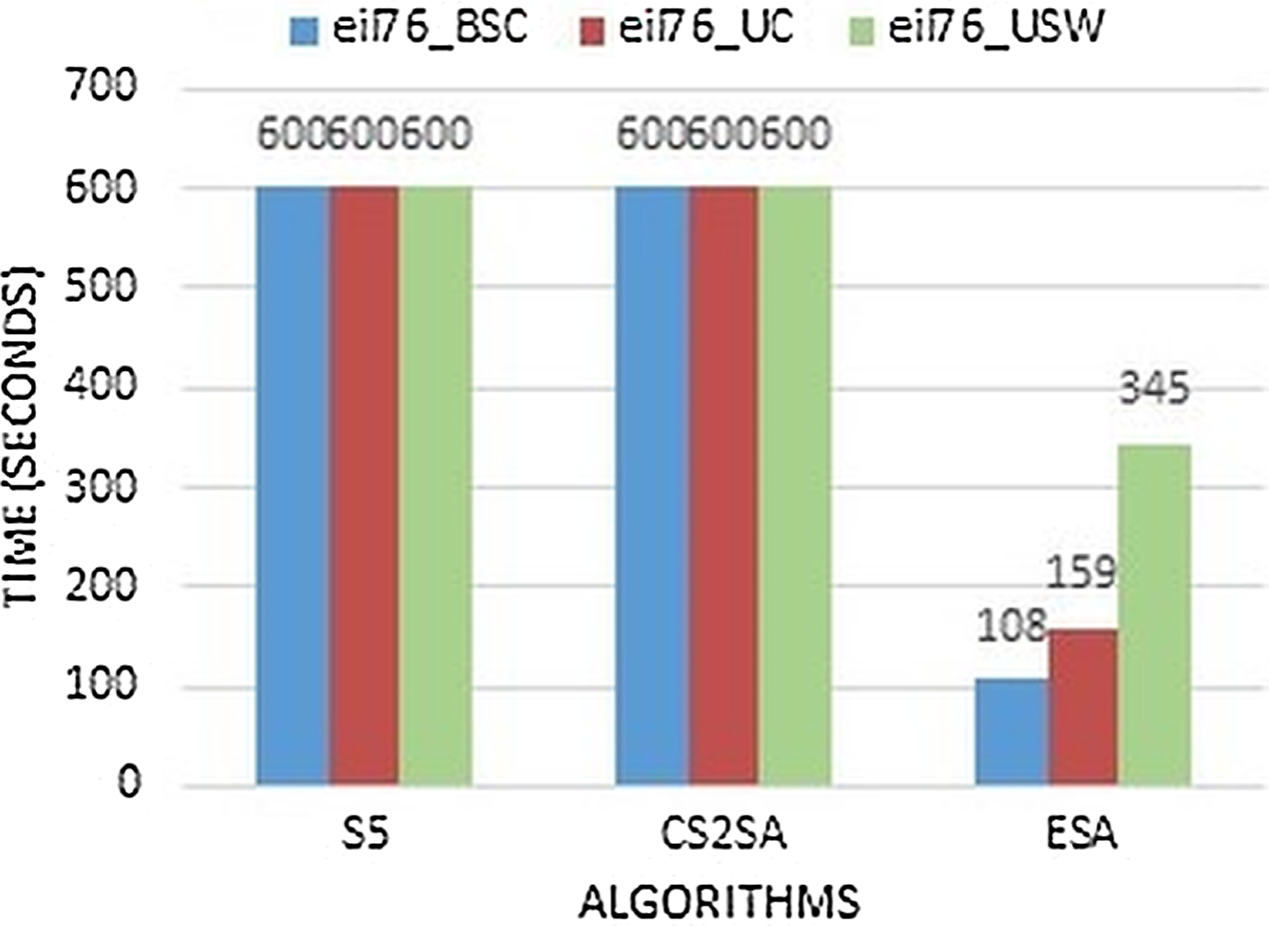
Time comparison of instance eil76.

The existing algorithms perform less than ESA and take maximum time to solve this problem. So, in small instances, this study concluded that ESA performs better in most instances and gives much efficiency in all instances. These instances are compared with execution time as presented in Fig. 4. It performs better results in few seconds as previous techniques perform this task in 600 s but ESA performs in 108, 159 and 345 s respectively.

**Figure 5:**
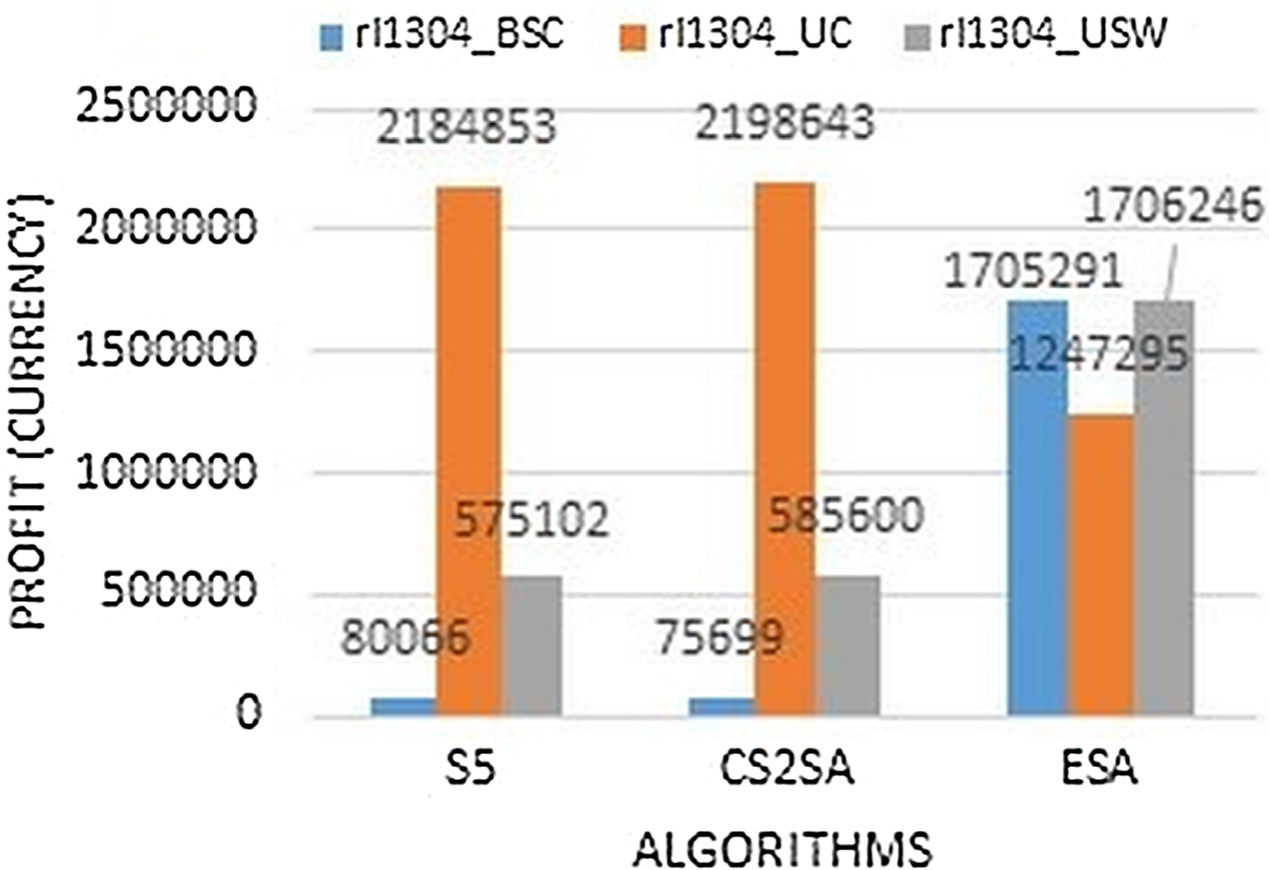
Results comparison of instance rl1304.

The Fig. 5 clearly demonstrates that the bounded correlated strongly instance perform much better in ESA as it gains 1,705,291, but in an uncorrelated instance, the performance degrades and in an uncorrelated similar weight, it also performs much better as it gains 1,706,246 profit. So in the instance of rl1304, two types of instance perform much better with this technique.

**Figure 6:**
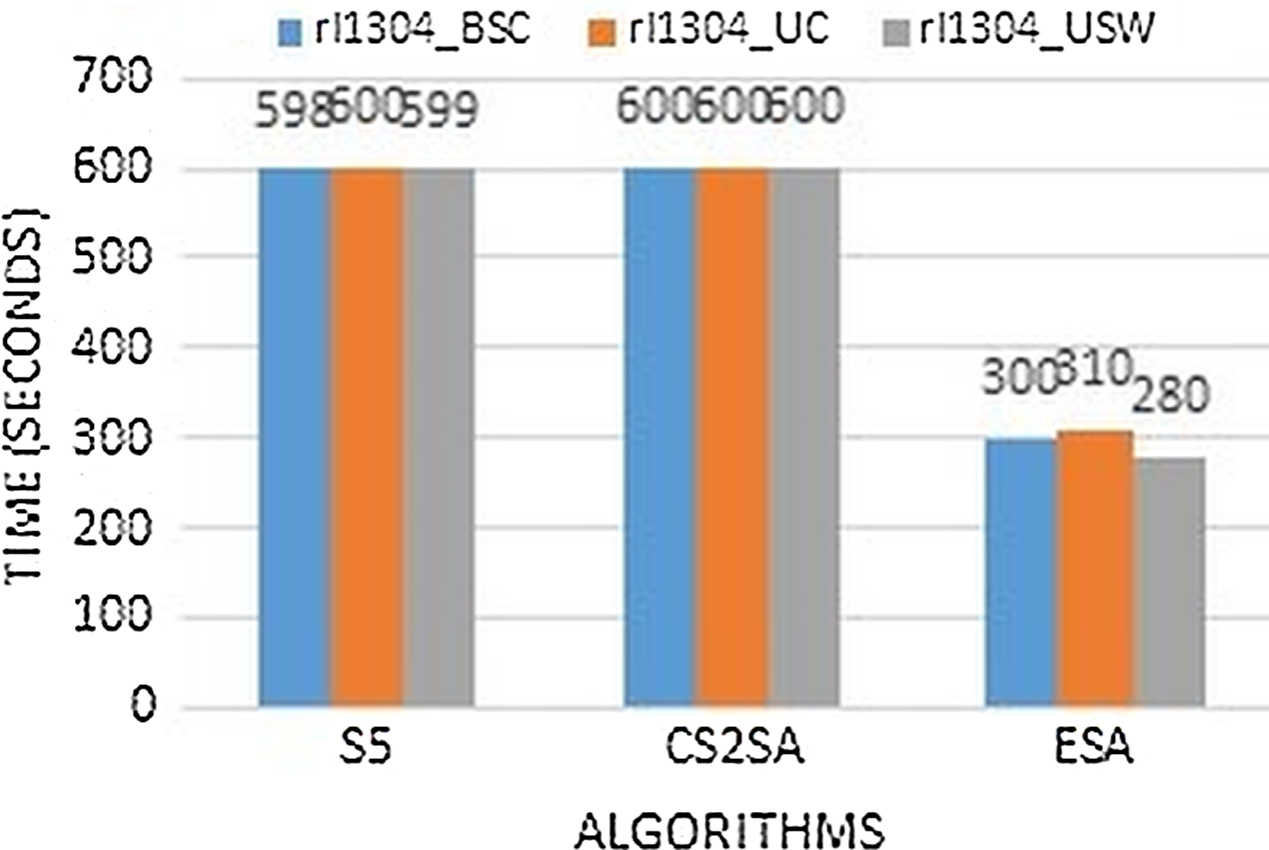
Time comparison of instance rl1304.

The Fig. 6 clearly describe that performance is much better than existing algorithms by comparing the execution time. Instance rl1304 performs better results in few seconds as previous techniques perform this task in 600 s but ESA performs in only 300, 310 and 280 s respectively.

**Figure 7:**
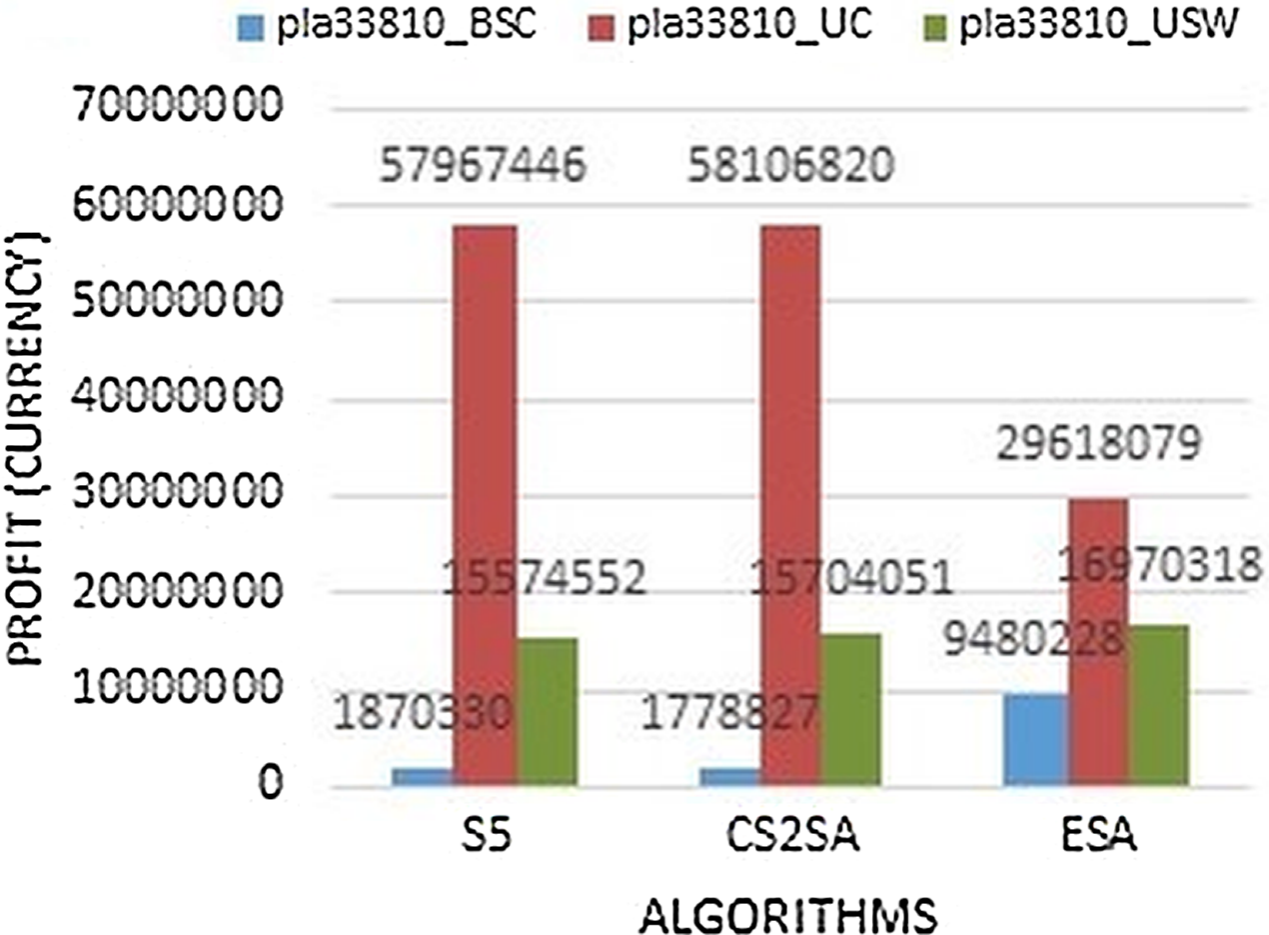
Results comparison of instance pla33810.

Large instances focus in most of the studies. In this paper, the instances which are included large instances having more than 15,000 number of cities. The Fig. 7 shows that, all the results perform better in all instances as compared to two other state-of-the-art algorithms. Two files perform better with ESA and performance degrade in the only uncorrelated file which gains 29,618,079 profit.

**Figure 8:**
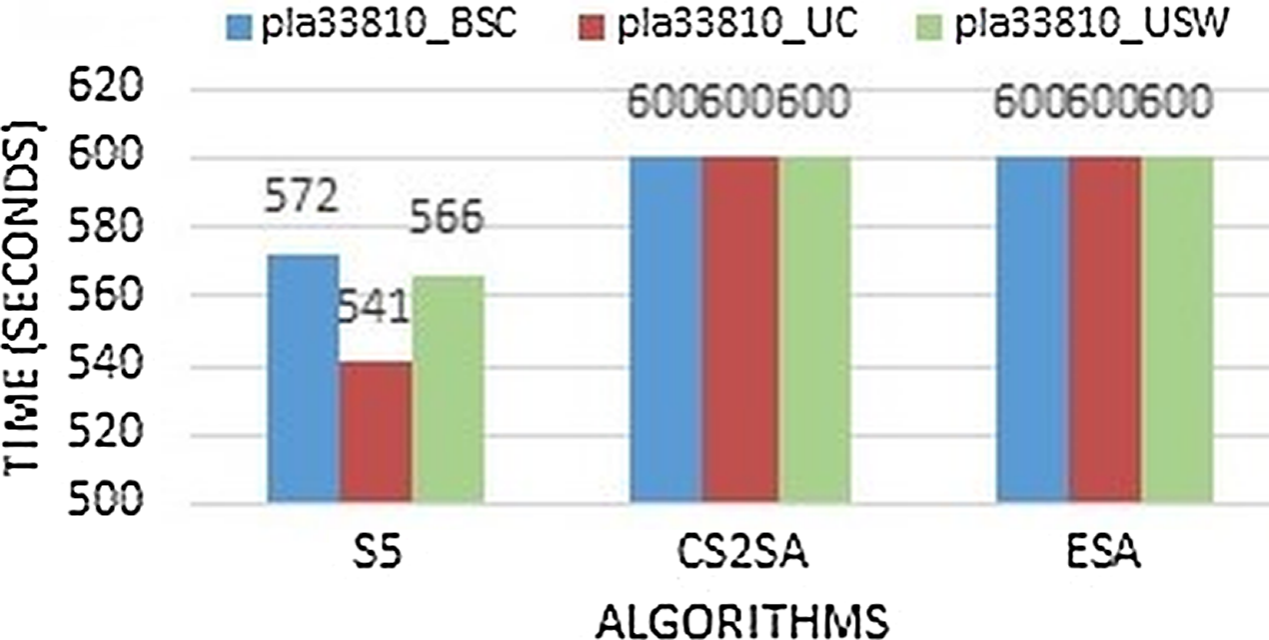
Time comparison of instance pla33810.

The Fig. 8 shows that all instances solve in a maximum time limit. So, in large instances, this study concluded that ESA performs better and competitive in most instances but it takes the maximum time to execute the file. Moreover, the more time is taken but the profit of these instances covers up the execution time of the instance as it gives more profit.

Table 3 clearly demonstrates the overall performance of our technique in which different experiments are performed with three associate instances with diverse weights. These sub-instances are stated above as bounded strongly correlated, uncorrelated with similar weights and uncorrelated (Bonyadi, Michalewicz & Wagner, 2014). Our algorithm clearly shows that the results mentioned in Table 2 perform much better by eliminating the cities on which we cannot get any profit. This is performed by analyzing that when we don’t take an item from city x then why we have to travel to such a city. This will overhead all cost traveling from city *X*_*i*_ to *X*_*i*+1_. As remember our knapsack is rented and has many burdens to travel from one city to another. So, we eliminate that city to improve the profit. As the profit will increase the cost of traveling will decease automatically because they both are inversely proportional to each other. Moreover, time will also decrease here to solve the TTP.

**Table 3:** Overall comparison of results.

Instances	BSC	UC	USW
*eil*76	+	+	+
*kroA*100	+	+	–
*ch*130	+	–	+
*u*159	+	–	+
*a*280	+	–	+
*u*574	+	–	–
*u*724	+	–	+
*dsj*1000	+	–	+
*rl*1304	+	–	+
*fl*1577	+	–	+
*d*2103	+	–	+
*pcb*3038	+	+	–
*fnl*4461	+	+	+
*pla*7397	+	+	–
*rl*11849	+	–	+
*usa*13509	+	–	+
*brd*14051	+	–	+
*d*15112	+	+	+
*d*18512	+	+	–
*pla*33810	+	–	+

Here we have discussed the trend of different sizes to solve TTP with different techniques. As many optimization approaches are performed by many researchers to solve this problem. Many of them target the specific ranges that is, small, medium-size, large. In this approach, we have concluded that this approach performs significantly well for small and medium sizes and compatible with the large size instance (Mei, Li & Yao, 2014). By eliminating the non-benefit cities from the tour this technique differs on the large size and gives better objective value as we are targeting to maximize the profit and minimize the cost and time as well. However, we have faith that as the tour is generated randomly, it will give better results if the tour and picking plan is focused more deeply and total time travel is also reduced in many instances.

## Conclusion

In this article, we have proposed a technique in which simulated annealing is modified to solve the TTP. This problem contains a person known as a thief and plans a tour to collect multiple items to fill his knapsack to gain maximum profit and minimum cost in 600 s as a standard of TTP time duration. This technique efficiently solves the TTP problem by rearranging the steps; first it creates a picking plan, and then generates a tour. This tour is then improved by eliminating the cities which are useless. The proposed technique on different instances shows promising results as compared to other states-of-the-art algorithms. We have computed many different instances and mentioned many of them which are selected and compared, in which many instances show that the result outperforms and rather they are competitive. So, at this stage, we may get the infinity value as a profit.

Future research will be focusing on multiple aspects of the knapsack problem, including enhancing the city elimination process. More work is needed to enhance the capability of the proposed approach towards larger instances.

## Supplemental Information

10.7717/peerj-cs.377/supp-1 Supplemental Information 1 Code and data.

10.7717/peerj-cs.377/supp-2 Supplemental Information 2 Comparison of instance brd14051.

10.7717/peerj-cs.377/supp-3 Supplemental Information 3 Comparison of instance pla7397.

10.7717/peerj-cs.377/supp-4 Supplemental Information 4 Times Comparison of instance pla7397.

10.7717/peerj-cs.377/supp-5 Supplemental Information 5 Comparison of instance ch130.

10.7717/peerj-cs.377/supp-6 Supplemental Information 6 Times Comparison of instance brd14051.

10.7717/peerj-cs.377/supp-7 Supplemental Information 7 Times Comparison of instance ch130.

10.7717/peerj-cs.377/supp-8 Supplemental Information 8 Comparison of instance pcb.

10.7717/peerj-cs.377/supp-9 Supplemental Information 9 Comparison of instance d2103.

10.7717/peerj-cs.377/supp-10 Supplemental Information 10 Times Comparison of instance pcb.

10.7717/peerj-cs.377/supp-11 Supplemental Information 11 Times Comparison of instance d2103.

10.7717/peerj-cs.377/supp-12 Supplemental Information 12 Comparison of instance u159.

10.7717/peerj-cs.377/supp-13 Supplemental Information 13 Times Comparison of instance u574.

10.7717/peerj-cs.377/supp-14 Supplemental Information 14 Comparison of instance u574.

10.7717/peerj-cs.377/supp-15 Supplemental Information 15 Times Comparison of instance u159.

10.7717/peerj-cs.377/supp-16 Supplemental Information 16 Comparison of instance u724.

10.7717/peerj-cs.377/supp-17 Supplemental Information 17 Times Comparison of instance usa13509.

10.7717/peerj-cs.377/supp-18 Supplemental Information 18 Times Comparison of instance u724.

10.7717/peerj-cs.377/supp-19 Supplemental Information 19 Comparison of instance usa13509.

## Additional Information and Declarations

### Data Availability

The following information was supplied regarding data availability: The code and data are available in the Supplemental Files. TTP instances are available at The University of Adelaide: https://cs.adelaide.edu.au/~optlog/CEC2014COMP_InstancesNew/.
