# Supplementary figures and images for: A novel approach for solving travelling thief problem using enhanced simulated annealing
Source: PeerJ Comput Sci. 2021 Mar 16;7:e377. doi: 10.7717/peerj-cs.377 (PMC8022508; doi:10.7717/peerj-cs.377)

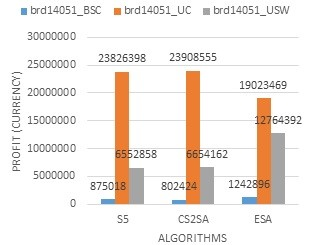

Supplement: Supplemental Information 2 [file peerj-cs-07-377-s002.png]

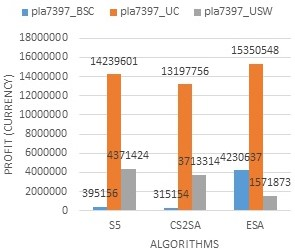

Supplement: Supplemental Information 3 [file peerj-cs-07-377-s003.png]

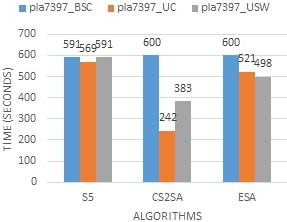

Supplement: Supplemental Information 4 [file peerj-cs-07-377-s004.png]

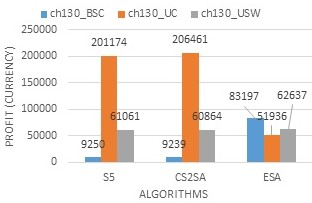

Supplement: Supplemental Information 5 [file peerj-cs-07-377-s005.png]

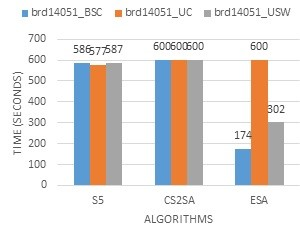

Supplement: Supplemental Information 6 [file peerj-cs-07-377-s006.png]

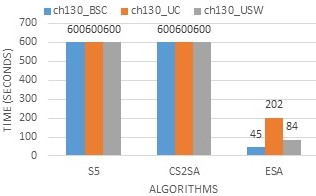

Supplement: Supplemental Information 7 [file peerj-cs-07-377-s007.png]

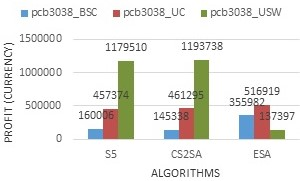

Supplement: Supplemental Information 8 [file peerj-cs-07-377-s008.png]

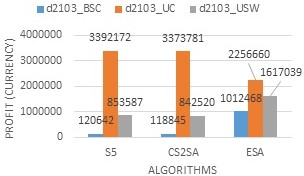

Supplement: Supplemental Information 9 [file peerj-cs-07-377-s009.png]

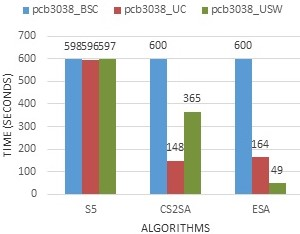

Supplement: Supplemental Information 10 [file peerj-cs-07-377-s010.png]

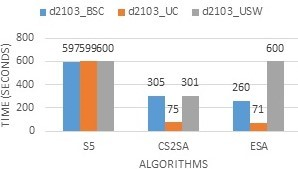

Supplement: Supplemental Information 11 [file peerj-cs-07-377-s011.png]

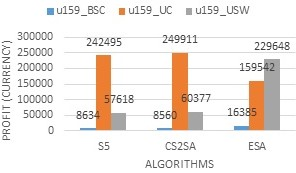

Supplement: Supplemental Information 12 [file peerj-cs-07-377-s012.png]

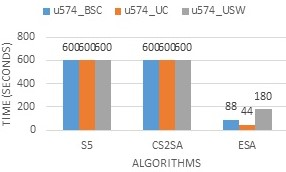

Supplement: Supplemental Information 13 [file peerj-cs-07-377-s013.png]

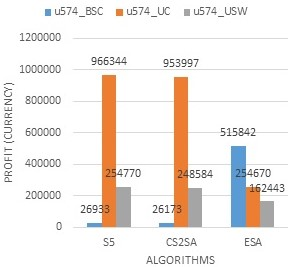

Supplement: Supplemental Information 14 [file peerj-cs-07-377-s014.png]

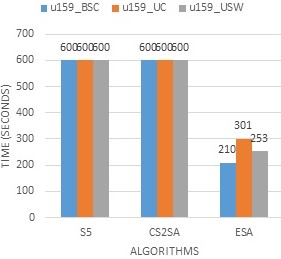

Supplement: Supplemental Information 15 [file peerj-cs-07-377-s015.png]

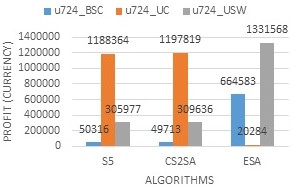

Supplement: Supplemental Information 16 [file peerj-cs-07-377-s016.png]

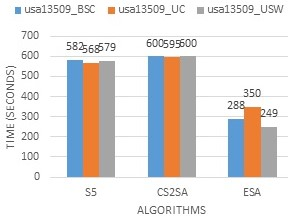

Supplement: Supplemental Information 17 [file peerj-cs-07-377-s017.png]

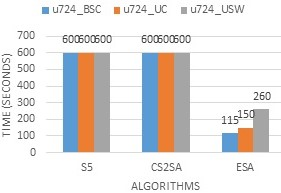

Supplement: Supplemental Information 18 [file peerj-cs-07-377-s018.png]

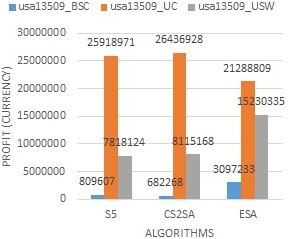

Supplement: Supplemental Information 19 [file peerj-cs-07-377-s019.png]
